# Bucky Ball Is a Novel Zebrafish Vasa ATPase Activator

**DOI:** 10.3390/biom11101507

**Published:** 2021-10-13

**Authors:** Roshan Priyarangana Perera, Alaa Shaikhqasem, Nadia Rostam, Achim Dickmanns, Ralf Ficner, Kai Tittmann, Roland Dosch

**Affiliations:** 1 Department of Developmental Biochemistry, University of Goettingen, 37077 Goettingen, Germany; r.dosch@kabelmail.de; 2 Department for Molecular Structural Biology, University of Goettingen, 37077 Goettingen, Germany; alaa.shaikhqasem@biochemtech.uni-halle.de (A.S.); adickma@gwdg.de (A.D.); rficner@uni-goettingen.de (R.F.); 3 Institute for Human Genetics, University of Goettingen, 37073 Goettingen, Germany; nadia.rostam@univsul.edu.iq; 4 deCluster of Excellence “Multiscale Bioimaging: From Molecular Machines to Networks of Excitable Cells” (MBExC), University of Goettingen, 37073 Goettingen, Germany; 5 Department of Molecular Enzymology, University of Goettingen, 37077 Goettingen, Germany; Kai.Tittmann@biologie.uni-goettingen.de

**Keywords:** germline, germplasm, zebrafish, *Drosophila*, primordial germ cells, Bucky ball, Oskar, Vasa, ATPase activity

## Abstract

Many multicellular organisms specify germ cells during early embryogenesis by the inheritance of ribonucleoprotein (RNP) granules known as germplasm. However, the role of complex interactions of RNP granules during germ cell specification remains elusive. This study characterizes the interaction of RNP granules, Buc, and zebrafish Vasa (zfVasa) during germ cell specification. We identify a novel zfVasa-binding motif (Buc-VBM) in Buc and a Buc-binding motif (zfVasa-BBM) in zfVasa. Moreover, we show that Buc and zfVasa directly bind in vitro and that this interaction is independent of the RNA. Our circular dichroism spectroscopy data reveal that the intrinsically disordered Buc-VBM peptide forms alpha-helices in the presence of the solvent trifluoroethanol. Intriguingly, we further demonstrate that Buc-VBM enhances zfVasa ATPase activity, thereby annotating the first biochemical function of Buc as a zfVasa ATPase activator. Collectively, these results propose a model in which the activity of zfVasa is a central regulator of primordial germ cell (PGC) formation and is tightly controlled by the germplasm organizer Buc.

## 1. Introduction

The germline is an essential feature of life that ensures the perpetuation and survival of a species by passing the genetic information from one generation to the next by specialized germ cells [1,2,3]. In many metazoan organisms, primordial germ cells (PGCs) are segregated from somatic cells during early embryonic development [4]. In mammals such as, mice and humans, a subset of pluripotent embryonic cells is specified to PGCs by cell–cell communication (induction mode) through zygotic signaling molecules [1,4,5,6,7,8]. Non-mammalian species such as *Drosophila*, *C. elegans*, *Xenopus*, and zebrafish require a specialized maternal cytoplasm referred to as germplasm to form PGCs (inherited mode) [9]. The germplasm is inherited by a set of blastomeres to change their fate into PGCs [1,4,5,6,7,8]. Studies carried out on the ablation, transplantation, and mislocalization of germplasm during embryonic development have revealed that germplasm is sufficient and necessary for the formation of PGCs in *Xenopus*, *Drosophila*, and zebrafish [10,11,12,13,14,15].

In *Drosophila*, the Oskar (Osk) protein is sufficient and necessary for the formation of germplasm and PGC specification [16,17,18,19]. Similarly, we discovered Bucky ball (Buc) as the first vertebrate-specific protein, which performs the same molecular functions in zebrafish [20,21,22]. Fascinatingly, we observed that the overexpression of *Drosophila* short Oskar (sOsk: amino acids 139–606) induces ectopic PGCs in zebrafish, even though sOsk shares no sequence homology with Buc. This phenomenon revealed, for the first time, a functional equivalence of two germplasm organizers [23]. Therefore, it seems that the molecular mechanisms of the PGC specification pathway are likely to be evolutionarily conserved between *Drosophila* and zebrafish.

Recent studies demonstrated that the LOTUS domain of Osk physically binds to the C-terminal RecA-like domain (RecA-CTD) of *Drosophila* Vasa (DmVasa), which stimulates its ATPase activity [24,25]. We also showed that Buc interacts with zebrafish Vasa (zfVasa) in vivo during germ cell specification [23]. Besides, we observed that the overexpression of zfVasa induced ectopic germ cells, identifying zfVasa as the second protein with a germ cell specification activity in zebrafish [23]. However, thus far, a zfVasa-binding motif in Buc (Buc-VBM) or Buc-binding motif in zfVasa (Vasa-BBM) has not been identified. Moreover, no specific biochemical function could be ascribed for Buc apart from being recognized as a scaffolding protein that brings proteins together, such as Hermes, Tdrd6a, or Igf2bp3 [26,27,28]. The biochemical function of Buc-specifying PGCs remains to be defined.

In this study, we characterized the Buc and zfVasa interaction on the protein level during germ cell specification. We identified a novel zfVasa-binding motif in Buc (Buc-VBM) and a Buc-binding motif in zfVasa (zfVasa-BBM) using bimolecular fluorescence complementation (BiFC assay) [29]. We further demonstrate that Buc-VBM physically binds to zfVasa in vitro. Based on the computational prediction, we illustrate that Buc-VBM encodes an intrinsically disordered peptide. Besides, our circular dichroism (CD) spectroscopy data revealed that the Buc-VBM adopts α-helices from its disordered state in the presence of cosolvent trifluoroethanol (TFE) that facilitates the secondary structure formation of peptides and proteins. Finally, we show that Buc-VBM stimulates the enzymatic ATPase activity of zfVasa in vitro, annotating the first biochemical function to Buc as a novel cofactor, which stimulates the ATPase activity of zfVasa. Thus, our data suggest that Buc interacts with the RNA helicase Vasa during PGC specification using similar structural changes during the interaction to perform equivalent functions as Oskar in *Drosophila*.

## 2. Materials and Methods

### 2.1. Zebrafish Handling and Maintenance

Experiments were performed using AB*TLF zebrafish (wild-type) and the Buc-GFP transgenic zebrafish line [30]. All the fish were maintained and fed according to the standard protocol [31].

### 2.2. Microinjection

The injections were performed as previously described [22]. The capped sense RNA was diluted in nuclease-free water and 0.025–0.05% phenol red and 0.1-M KCl. At least 30 embryos were sorted for microinjection. One-cell stage embryos were injected with 1 nL of diluted RNA containing 150 ng/μL of each candidate RNA.

### 2.3. BiFC Assay

The BiFC assay was performed as described previously [29]. Briefly, the BiFC assay is based on the reconstitution of an intact Venus fluorescent protein complex when two complementary nonfluorescent fragments of a Venus protein are brought in close proximity when two proteins interact upon their expression in living cells. In this study, the Venus C-terminal half is fused to the C-terminal region of full-length of Buc and truncated Buc constructs while the Venus N-terminal half is fused to the C-terminal region of full-length zfVasa and truncated zfVasa constructs. Wild-type embryos were injected at the one-cell stage with the mRNA constructs (200 ng/μL). The injected embryos were sorted and imaged for fluorescence at the 3 h post-fertilization stage (hpf) with a LSM780 confocal microscope (Carl Zeiss Microscopy, Jena, Germany).

### 2.4. Live Cell Imaging

The embryos were manually dechorionated at the 3 hpf stage and mounted on 1.5% agarose-coated dishes filled with 1x E3 medium and imaged at the same stage by a stereo microscope SteREO Lumar.V12 (Carl Zeiss Microscopy, Jena, Germany). The images were processed using the software Axio Vision Rel. 4.8 (Carl Zeiss Microscopy, Jena, Germany). For imaging with LSM80, dechorionated embryos were placed on a Fluorodish (WPI, Sarasota, FL, USA) with 1x E3. The images were analyzed using ZEN 2011 software (Carl Zeiss Microscopy, Jena, Germany).

### 2.5. Quantification of Fluorescence Intensity

The images were uploaded into ImageJ software (ImageJ bundled with 64-bit Java 1.8.0_172) [32]. The fluorescence intensity was calculated after highlighting the margin of the blastodisc of the embryos.

### 2.6. Recombinant Protein Expression of Buc-VBM

The Buc-VBM encoding amino acids 363–400 was amplified by PCR. The PCR product was cloned into the pGEX-6P1 vector, which contains a GST fusion tag using the *EcoRI* restriction enzyme. The GST fusion protein was expressed in BL21 (DE3) cells. The protein expression was induced by adding IPTG to a 0.5-mM final concentration and incubated overnight at 16 °C while shaking at 200 rpm. Cells were harvested and resuspended in lysis buffer. Cells were disrupted using a microfluidizer (Microfluidics, Westwood, MA, USA) in 50-mM Tris/HCl (pH 7.8), 500-mM NaCl, 5% (*v*/*v*) glycerol, and 10-mM ethylenediaminetetraacetic acid (EDTA) + 1 protease inhibitor cOmplete ULTRA tablet/50-mL buffer). The lysate was clarified by ultracentrifugation at 30,000× *g* and 4 °C for 30 min. The clarified lysate was loaded on a glutathione Sepharose column (GE healthcare, Chicago, IL, USA) at room temperature. The protein was subsequently eluted with 30-mM reduced glutathione. The GST tag was proteolytically cleaved by adding PreScission Protease (1 µL (2 units) per 100 µg of fusion protein) to the eluted protein and incubating it overnight at 4 °C. The tag was removed using a Superdex 75 gel filtration column coupled to a glutathione Sepharose column (GE healthcare, Chicago, IL, USA) in 20-mM Tris/HCl (pH 7.8), 200-mM NaCl, 5% glycerol, and 2-mM MgCl_2_. The protein was concentrated by an Amicon Ultra centrifugal concentrator (Merck, Darmstadt, Germany) and flash-frozen in liquid nitrogen.

### 2.7. Recombinant Protein Expression of zfVasa (227–670) aa

The zfVasa encoding amino acids 227–670 was amplified by PCR. The PCR product was cloned into the pGEX-6P1 vector, which contains a GST fusion tag using the *BamHI* restriction enzyme. The GST fusion protein was expressed in BL21 (DE3) cells. The protein expression was induced, adding IPTG to the 0.5-mM final concentration, together with 4% ethanol (*v*/*v*) and K_2_HPO_4_ to a 30-mM final concentration, and incubating it overnight at 16 °C while shaking at 200 rpm. Cells were harvested and resuspended in lysis buffer. Cells were disrupted using a microfluidizer (Microfluidics, Westwood, MA, USA) in 50-mM Tris/HCl (pH 7.8), 500-mM NaCl, 5% (*v*/*v*) glycerol, and 10-mM ethylenediaminetetraacetic acid (EDTA) + 1 protease inhibitor cOmplete ULTRA tablet/50-mL buffer). The lysate was clarified by ultracentrifugation at 30,000× *g* and 4 °C for 30 min. The clarified lysate was loaded on a glutathione Sepharose column (GE healthcare, Chicago, IL, USA) at room temperature. Potentially bound nucleic acids were removed by washing the lysate with wash buffer supplemented with 2-M LiCl. The protein was subsequently eluted with 30-mM reduced glutathione. The GST tag was proteolytically cleaved by adding PreScission Protease (1 µL (2 units) per 100 µg of fusion protein) to the eluted protein and incubating it overnight at 4 °C. The tag was removed using a Superdex 75 gel filtration column coupled to a glutathione Sepharose column (GE healthcare, Chicago, IL, USA) in 20-mM Tris/HCl (pH 7.8), 200-mM NaCl, 5% glycerol, and 2-mM MgCl_2_. The protein was concentrated by an Amicon Ultra centrifugal concentrator (Merck, Darmstadt, Germany) and flash-frozen in liquid nitrogen.

### 2.8. Coomassie Staining

After SDS-gel electrophoresis, the gels were washed with dH_2_O. Thereafter, the gels were incubated in Coomassie staining solution (50% methanol, 10% glacial acetic acid, and 0.1% Coomassie Brilliant Blue) for 30 min to 1 h at room temperature. The stained gels were washed three times with dH_2_O and incubated in a destaining solution (40% methanol and 10% glacial acetic acid) overnight at room temperature.

### 2.9. GST Pull-Down Assay

In the pull-down assay, 0.5 nmol of GST-Buc-VBM (amino acids 363–400) was incubated with 50 μL of glutathione Sepharose^TM^ 4B (GE Healthcare) beads in a pull-down buffer (20-mM Tris, pH 7.5, 150-mM NaCl, 2-mM MgCl_2_, and 1-mM DTT) at 25 °C for 30 min. Later, 20 nmol of zfVasa (amino acids 227–670) was added to the mixture and incubated at 25 °C for another 30 min. Afterward, the beads were washed three times with 1 mL of pull-down buffer. Bound proteins were eluted in 20 μL of pull-down buffer supplemented with 30-mM reduced glutathione. After elution, the samples were centrifuged for 10 min at 20,000× *g* and at 4 °C. The supernatant was then carefully transferred into clean tubes, mixed with 2X SDS-PAGE loading dye, and analyzed by SDS-PAGE followed by Coomassie staining.

### 2.10. ATPase Assay

The ATPase activity of zfVasa was measured with a nicotinamide adenine dinucleotide (NADH)-dependent coupled enzymatic assay [33]. The assay detects the reduction of the NADH absorption at 340 nm as a direct effect of ATP consumption over time with a VICTOR Nivo multimode microplate reader (PerkinElmer, Waltham, MA, USA). The ATPase activity of zfVasa was determined by mixing 5-µM zfVasa with and without 100 µM of Buc-VBM, together with 2.5-mM ATP, 250-nM NADH, 500-nM phosphoenolpyruvate, 6–8.3-U/mL pyruvate kinase, and 9–14-U/mL lactic dehydrogenase. The RNA-stimulated ATPase activity of zfVasa was performed by adding 50-µM ssRNA (polyA_(8)_) into 5-µM zfVasa with and without 100 µM of Buc-VBM with the other common components listed. All reactions were performed in triplicates of 150 µL in each at 25 °C.

### 2.11. Circular Dichroism (CD) Spectroscopy

The CD spectroscopic analysis of Buc-VBM (amino acid 363–400) was carried out using a Chirascan CD spectrometer (Applied Photophysics, Leatherhead, UK) in the far-UV (185–260 nm). Initially, the protein buffer was exchanged to a buffer containing 20-mM Na-phosphate buffer to minimize the buffer signals. Measurements were recorded at room temperature using a concentration of 0.1-mg/mL Buc-VBM. For each sample, 15 individual spectra were recorded with a time-per-point value of 1 s and an optical path length of 1 mm. The collected spectra were averaged, and the spectra values for the sample buffer were subtracted. The CD data are presented in units of mean residue weight molar ellipticity ((θ)deg × cm^2^/dmol) and plotted using QtiPlot (v.0.9.8.9) application.

### 2.12. Bioinformatics Methods

The sequences were pairwise aligned using the Needleman-Wunsch algorithm (https://www.ebi.ac.uk/Tools/psa/emboss_needle/). Multiple sequence alignment was performed using the T-Coffee multiple sequence alignment server (https://www.ebi.ac.uk/Tools/msa/tcoffee/) [34]. The protein sequences were aligned using the T-Coffee Expresso server (http://tcoffee.crg.cat/apps/tcoffee/do:expresso) [35]. Further, in silico protein modeling: PyMol Ve 2.3 application was used to visualize and for the structural arrangement and alignment of the predicted protein model. Homology modeling: The homology model of zfVasa was predicted using the I-Tasser protein structure and function predictions server (https://zhanglab.dcmb.med.umich.edu/I-TASSER/).

### 2.13. Statistics

All the statistical analyses were performed using GraphPad Version 8. All the samples used in this study consisted of three independent sample groups. Two-tailed *t*-test and one-way ANOVA were used as the test statistics. The significance levels were considered as * = 0.05, ** = 0.01, *** = 0.001, and **** = 0.0001. Errors represent the standard deviation.

## 3. Results

### 3.1. Identification of the zfVasa-Binding Motif in Buc (Buc-VBM)

**Figure 1:**
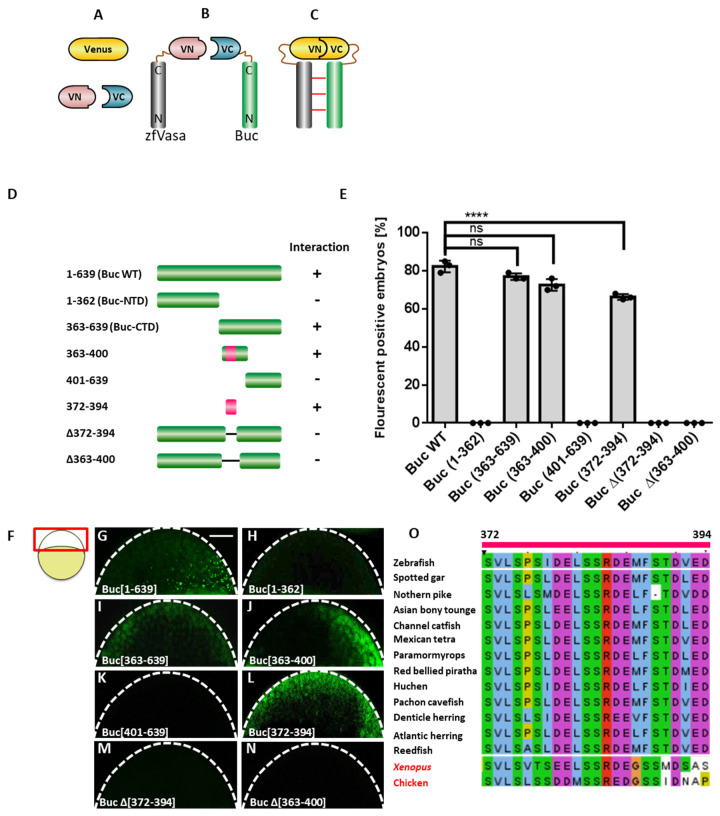
Mapping of the Vasa-binding motif in Buc (Buc-VBM). (**A**–**C**) Illustration of the BiFC assay. (**A**) Venus protein (yellow) is split into two nonfluorescent parts, Venus N-terminus (VN; rose) and Venus C-terminus (VC; blue) (**B**) Fusion of zfVasa (grey) and Buc (green) with VN and VC fragments, respectively. (**C**) Interaction of zfVasa and Buc (horizontal red lines) reconstitute a functional Venus protein forming a bimolecular fluorescent complex. (**D**) Schematic illustration of the systematic truncation of Buc (green). Numbers left to the colored bars indicate the corresponding amino acids. Interactions are marked by a ‘+’, while no interactions as ‘-’. Buc-VBM is indicated in pink. (**E**) Quantification of the fluorescent-positive embryos based on different combinations of the BiFC Buc and zfVasa constructs. The data presented are averaged from three independent experiments. The Y-axis represents a percentage of the average fluorescent embryos, and the X-axis represents injected constructs. (**G**–**N**) Confocal images of live embryos at 3 hpf (hours post-fertilization) after the injection of Buc constructs with wild-type zfVasa. The imaging area is boxed in red, as indicated in the cartoon on the left (**F**). This region is outlined with a white dashed line (**G**–**N**). The injection of wild-type Buc showed a fluorescent signal (**G**; 82 ± 3.9%, *n* = 58). After splitting Buc into Buc-NTD (amino acids 1–362) and Buc-CTD (amino acids 363–639), only the Buc-CTD show fluorescence (**I**; 77 ± 5.7%, *n* = 79), and there is no florescence with Buc-NTD (**H**; 0 ± 0%, *n* = 105). Splitting Buc-CTD into two parts, the construct containing the amino acids 363–400 showed fluorescent embryos (**J**; 72 ± 4.1%, *n* = 79) but not the amino acids 401–639 (**K**; 0 ± 0%, *n* = 77). Interestingly, the highly conserved domain of Buc (amino acids 372–394) displayed fluorescence (**L**; 66 ± 2.8%, *n* = 69). No fluorescent signal was observed after deleting amino acids 372–394 in full-length Buc (BucΔ (372–394) (**M**; 0 ± 0%, *n* = 71) and BucΔ (363–400) (**N**; 0 ± 0%, *n* = 64). (**O**) Multiple sequence alignment revealed that amino acids 372–394 (labeled on the top of the sequence) are highly conserved in the Buc homolog in *Xenopus* and the Xvelo and Buc homologs in chickens, in addition to Buc orthologs in other teleost species. Amino acids are colored based on the ClustalX color code. Hydrophobic amino acids are (Alanine (A), Isoleucine (I), Leucine (L), Methionine (M), Phenylalanine (F), Tryptophan (W), and Valine (V)) colored in blue. Positively charge amino acids ((Lysine (K) and Arginine (R)) are colored in red. Negatively charge amino acids ((Aspartic acid (D) and Glutamic acid (E)) are colored in magenta. Polar amino acids ((Asparagine (N), Glutamine (Q), Serine (S), and Threonine (T)) are colored in green. Aromatic amino acids ((Histidine (H) and Tyrosine (Y)) are colored in cyan. Cystine is colored in pink. Glycine is colored in orange. Proline is colored in yellow. Protein sequences for Buc orthologs were retrieved from the Ensembl database (Ensembl Release 104), except the Buc homolog in *Xenopus* and the Xvelo and Buc homologs in chickens, which were retrieved from Xenobase Version 5.3.0 and the NCBI protein database, respectively [38,39]. The protein sequences used for multiple sequence alignment were: ENSDARP00000125536; Zebrafish (*Danio rerio*), ENSLOCP00000015864; Spotted gar (*Lepisosteus oculatus*), ENSELUP00000012527; Northern pike (*Esox luciusI*), ENSSFOP00015022504; Asian bonytongue (*Scleropages formosus*), ENSIPUP00000003130; Channel catfish (*Ictalurus punctatus*), ENSAMXP00000053358; Mexican tetra (*Astyanax mexicanus-2*), ENSPKIP00000037210; Paramormyrops (*Paramormyrops kingsleyae* ),ENSPNAP00000033609; Red-bellied piranha (*Pygocentrus nattereri*),ENSHHUP00000031919; Huchen (*Hucho hucho*), ENSAMXP00005041590; Pachon cavefish (*Astyanax mexicanus-1*), ENSDCDP00000002250; Denticle herring (*Denticeps clupeoides*), ENSCHAP00000009659; Atlantic herring (*Clupea harengus*), ENSECRP00000007749; Reedfish (*Erpetoichthys calabaricus*), XB-GENE-5934753; Xenopus (*Xenopus laevis*), XP_040546153.1; and chicken (*Gallus gallus*). Test statistics: Student’s *t*-test, **** = 0.0001. ns. = nonsignificant. Error bars represent the standard deviation of the mean. Scale bar 100 µm.

The amino acid sequence predicts Buc to be an intrinsically disordered protein (IDP) [23] (Supplementary Figure S1). Thus, after expressing the recombinant protein in *E. coli*, we found Buc in the insoluble pellet fraction concealing the chance to reveal its structural features and biochemical functions. Alternatively, isolating a small motif, which interacts with zfVasa, provides a promising approach to discover structural features and biochemical functions of the Buc-zfVasa interaction. To that end, we performed a bimolecular fluorescence complementation (BiFC) assay to determine the Buc-VBM (Figure 1A–C). The BiFC assay is based on the reconstitution of an intact fluorescent protein complex, if two interacting proteins bring two complementary intrinsically nonfluorescent fragments into close proximity upon their expression in living cells [29,36,37].

Initially, the Buc protein sequence (amino acids 1–639) was truncated into N-terminal (Buc-NTD: amino acids 1–362) and C-terminal (Buc-CTD: amino acids 363–639) halves (Figure 1D). The Venus C-terminal (VC) half was fused to the C-terminus of full-length of Buc (Buc-VC) and to the truncated Buc protein fragments (Figure 1B). The Venus N-terminal (VN) half was fused to the C-terminus of the full-length of zfVasa protein (zfVasa-VN) (Figure 1B). After a co-injection of mRNA encoding the respective Buc constructs and zfVasa-VN into one-cell stage zebrafish embryos, we observed that Buc-VC, as well as Buc-CTD (amino acids 363–639), show fluorescent aggregation with zfVasa (Figure 1E,G,I). We previously found an interaction of zfVasa with Buc (1–361) in vitro. However, we could not confirm this interaction in vivo [23]. In our results, the Buc-NTD (amino acids 1–362) did not show fluorescent aggregation (Figure 1E,H). We further truncated the Buc-CTD into two smaller nonoverlapping constructs: amino acids 363–400 and amino acids 401–639 (Figure 1D). Afterwards, we observed fluorescence only with the amino acids 363–400 of Buc (Figure 1E,J).

Previously, we identified a highly conserved sequence (amino acids 372–394) in Buc after sequence alignments of vertebrate Buc orthologs [23]. We directly examined whether this conserved sequence is directly involved in the interaction of Buc with zfVasa as the Buc construct encoding amino acids 363–400 encompasses this highly conserved sequence (Figure 1D). Indeed, we found that the Buc 372–394 amino acid sequence is sufficient to interact with zfVasa (Figure 1E,L). In the control, we deleted the amino acids 372–394 from full-length Buc (Buc Δ372–394) (Figure 1D). After the overexpression of BucΔ372–394, we did not detect any fluorescence in the embryos, suggesting that the protein section with amino acids 372–394 is sufficient and necessary for zfVasa interactions (Figure 1E,M). Therefore, for the first time, we established that the highly conserved region of Buc mediates the interaction with zfVasa, specifically defining amino acids 372–394 as the zfVasa-binding motif in Buc (Buc-VBM). We next checked whether the highly conserved Buc 372–394 amino acid sequence is conserved among Buc homologs in other vertebrates. Interestingly, we detected that this sequence is conserved in the chicken (*Gallus gallus)* homolog of Buc and in the *Xenopus* homolog of Buc, Xvelo (Figure 1O). Thus, we speculate that the chicken homolog of Buc and Xvelo are likely to bind Vasa during germ cell specification. Next, we aimed to purify the Buc-VBM (amino acids 372–394) for the downstream experiments. We realized, however, that the recombinant expression of a slightly longer Buc peptide (amino acids 363–400) proved more successful. Similar to BucΔ372–394, the BiFC assay of BucΔ363–400 with zfVasa also did not show fluorescence in the embryos (Figure 1D,E,N). Thus, we used the Buc 363–400 amino acid sequence for all subsequent experiments described in this paper.

### 3.2. Identification of the Buc Binding Motif in zfVasa (zfVasa-BBM)

**Figure 2:**
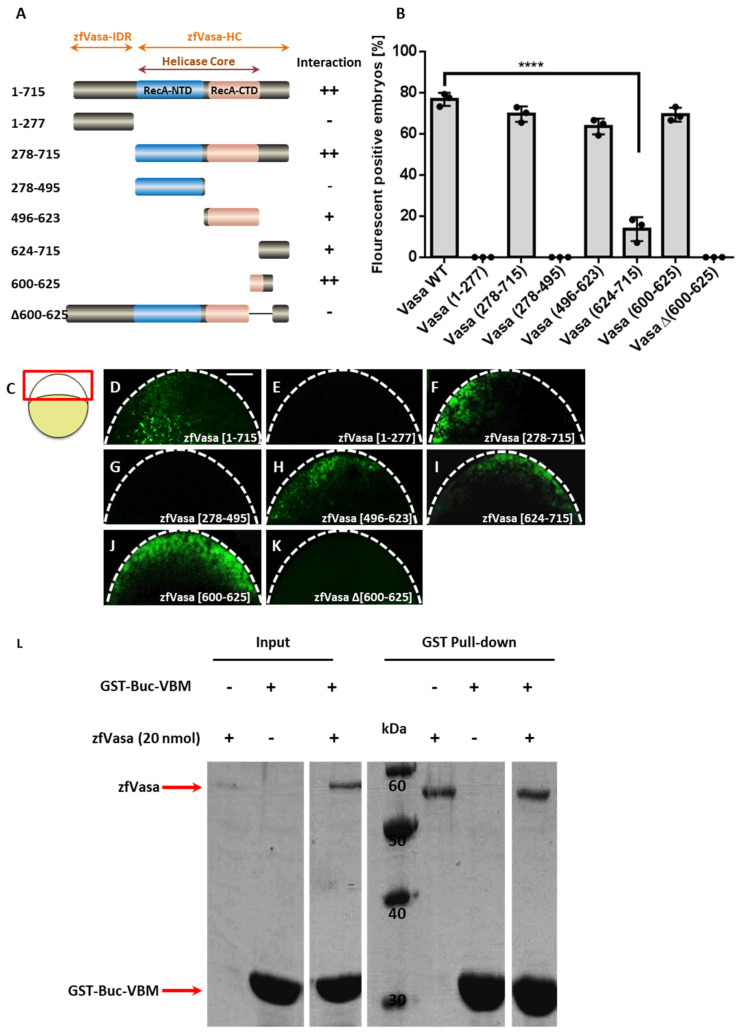
Mapping the Buc-binding motif in zfVasa (zfVasa-BBM). (**A**) Schematic illustration of the systematic truncation of zfVasa (dark grey) with a helicase core containing the N-terminal (blue) and C-terminal (light rose) RecA-like domains. Numbers left to the colored bars indicate the corresponding amino acids. (**B**) Quantification of fluorescent-positive embryos based on the microinjection of mRNA encoding the different combinations of BiFC Buc and zfVasa constructs. The data presented are averaged from three independent experiments. The Y-axis represents the percentage of fluorescent embryos, and the X-axis shows the injected constructs. Error bars represent the standard deviation of the mean. Average fluorescent-positive embryos ≥ 60% denoted as ‘++’, average fluorescent-positive embryos ≤ 60% shown as ‘+’, and embryos with no fluoresce denoted as ‘-‘. (**D**–**K**) Confocal images of live embryos at 3 hpf (hours post-fertilization) after the injection of zfVasa constructs with wild-type Buc. (**C**) The imaging area is boxed in red, as indicated in the cartoon on the left. This region is outlined with a white dashed line (**D**–**K**). Injection of wild-type zfVasa showed a fluorescent signal (**D**; 77 ± 3.0%, *n* = 96). After splitting zfVasa, zfVasa-IDR (amino acids 1–277 and labeled in orange on top of (**A**)) did not show fluorescence (E; 0 ± 0%, *n* = 43), but zfVasa-HC (amino acids 278–715 and labeled in orange on top of (**A**)) showed a fluorescent signal (F; 70 ± 4.0%, *n* = 85). From the three constructs of zfVasa-HC, the construct containing the amino acids 278–495 did not show an interaction signal (**G**; 0 ± 0%, *n* = 57). Besides, the construct containing the amino acids 496–623 showed more fluorescent embryos (**H**; 64 ± 4.0%, *n* = 76) than the other construct containing amino acids 624–715 (**I**; 14 ± 5.0%, *n* = 55). The zfVasa construct containing the amino acids 600–625 isolated a minimum peptide, which interacted with Buc (**J**; 69 ± 8.2%, *n* = 64). No fluorescent signal was observed after removing amino acids 600–625 in full-length zfVasa (zfVasaΔ (600–625)) (**K**; 0 ± 0%, *n* = 71). (**L**) SDS-PAGE (15%) stained with Coomassie Brilliant Blue. GST pull-down assay performed with recombinant GST-Buc-VBM (expected molecular weight approximately 32 kDa) and zfVasa (residues 227–670; expected molecular weight approximately 56 kDa). Protein markers (in kDa) are indicated in the middle. Compared to the control input samples (lanes 1–3), the pull-down (lanes 5–7) GST-Buc-VBM brings down zfVasa, suggesting that both fragments directly bind to each other. Scale bar 100 µm. Test statistics: Student’s *t*-test, **** = 0.0001. ns. = nonsignificant.

The first 277 amino acids of full-length zfVasa (amino acids 1–715) are predicted to be intrinsically disordered (zfVasa-IDR) (Figure 2A and Supplementary Figure S1), while residues 278–715, including the conserved helicase core, are likely structured (zfVasa-HC) (Figure 2A and Supplementary Figure S1). Until now, however, no protein structure has been experimentally determined for zfVasa. Nevertheless, we identified the essential domain features for zfVasa-HC from the GeneBank feature annotation provided for zfVasa (accession number: XP_005156510). Thus, we identified amino acids 278–495 as the RecA-like N-terminal domain (RecA-NTD) and amino acids 513–623 as the RecA-like C-terminal domain (RecA-CTD) (Figure 2A).

To map zfVasa-BBM (Buc-binding motif), we systematically truncated zfVasa. To this end, the Venus-N-terminal (VN) half was fused to the C-terminus of all the zfVasa constructs. First, we divided the zfVasa sequence into two halves, in which the first construct contains zfVasa-IDR (amino acids 1–277), and the other construct contains zfVasa-HC (amino acids 278–715) (Figure 2A). After co-injecting these two constructs with Buc-VC, we observed that only zfVasa-HC (amino acids 278–715) interacts with Buc-VC (Figure 2B,F). Interestingly, it has been described that the Osk LOTUS domain binds to RecA-CTD of DmVasa [24]. To find out whether Buc binds to zfVasa in a similar fashion, we tested if zfVasa-RecA-CTD can interact with Buc. To that end, we divided zfVasa-HC (amino acids 278–715) into three segments: (1) RecA-NTD (amino acids 278–495), (2) RecA-CTD (amino acids 496–623), and (3) the extended sequence (denoted as ‘e’) at the C-terminus of RecA-CTD (eRecA-CTD; amino acids 624–715) (Figure 2A). We detected that both RecA-CTD and eRecA-CTD constructs interact with Buc (Figure 2B,H,I), whereas RecA-NTD did not interact with Buc (Figure 2B,G). Although more embryos with fluorescence were observed with RecA-CTD (64%), we still observed some fluorescent embryos with eRecA-CTD (14%) (Figure 2B). Even though the reason for this interaction cannot be fully explained, we speculate that the truncation through the two domains likely perturbs the protein structure, which led to varying degrees of interactions. This would imply that the Buc interaction domain might be shared between the RecA-CTD and eRecA-CTD domains.

In order to further clarify this issue, we adapted the mapping strategy by removing 50 amino acids from the C-terminal region of the construct, containing both RecA-CTD and eRecA-CTD. While doing so, we detected that the number of fluorescent embryos dramatically dropped from 71% to 24% when we deleted 50 amino acids, trimming 665–615 (Supplementary Figure S2). This observation is similar to the results described above, where we observed a reduction of fluorescent embryos after splitting through RecA-CTD and eRecA-CTD (Figure 2B,H,I). Taken altogether, with this approach, we narrowed down zfVasa-BBM to residues 600–665. We then continued mapping by removing a further ten amino acids from the C-terminus of the previously identified construct (amino acids 600–665) (Supplementary Figure S2). Using this approach, we were able to identify amino acids 600–625 as the minimal sequence for zfVasa-BBM (Figure 2A,B,J). As a control, we deleted amino acids 600–625 from full-length zfVasa and checked for an interaction with Buc. We observed no fluorescent embryos, suggesting that residues 600–625 are indeed sufficient and necessary for zfVasa interactions (Figure 2A,B,K).

Nevertheless, this result is debatable, as the fluorescent signal produced in the BiFC assay does not always mirror a direct protein–protein interaction as the proximity of the nonfluorescent Venus fragment is a key requirement in the BiFC assay [29]. Thus, two candidate binding partners might emit fluorescence, although there is no direct physical interaction between two target proteins. Therefore, to further confirm a direct interaction between Buc and zfVasa, we performed in vitro GST pull-down assays. First, we purified GST-Buc-VBM (amino acids 363–400) and GST-zfVasa encoding amino acids 227–670, as this sequence showed a higher level of recombinant protein expression in the bacterial expression system. The purified GST-zfVasa was treated with PreScission Protease to remove the GST tag. Then, we performed the GST pull-down assay incubating GST-Buc-VBM with zfVasa. Our results showed that GST-Buc-VBM directly binds to zfVasa (Figure 2L). This confirms that the fluorescence signal that we observed during the BiFC assay is likely due to a direct interaction of Buc and zfVasa.

### 3.3. Buc-VBM Adopts α-Helices from Its Disordered Nature

**Figure 3:**
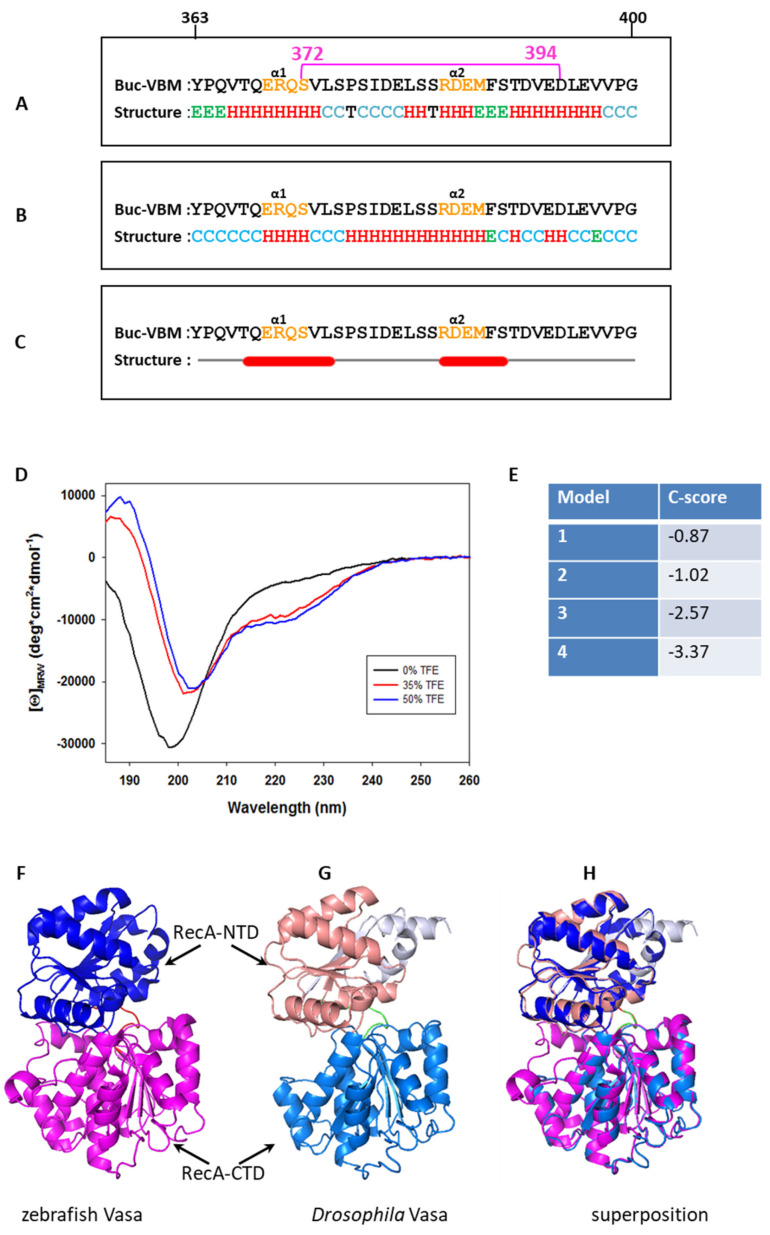
Buc-VBM adopts α-helices from its disordered nature in vitro. (**A**) In silico prediction of α-helices in Buc-VBM. In all three algorithms, the predicted α-helices are denoted as ‘H’ in red, β-sheets as ‘E’ in green, and turns as ‘T’ in black. The consensus secondary structure is shown at the bottom. Note that random coils in the consensus structure are denoted as ‘C’ in light blue. The first (363) and last (400) amino acids of Buc-VBM are displayed on top of the sequence (**A**–**C**). The highly conserved amino acids 372–394 are denoted by a pink, horizontal bar. (**A**) Secondary structure prediction using the CFSSP algorithm. The algorithm predicted two α-helices, α1 and α2. The α1-helix contains the amino acid sequence ERQS (orange), and α2 contains the amino acid RDEM (orange). CFSSP also predicted two β-sheets (green E), which contain the amino acids YPQ at the N-terminus and MFS at the C-terminus. (**B**) Secondary structure prediction using the PEP2D algorithm. The algorithm predicted two α-helices, α1 and α2, which share the same amino acids predicted by the CFSSP algorithm. (**C**) Secondary structure prediction using the Jnet algorithm. The algorithm predicted two α-helices, α1 and α2, which share the same amino acids predicted by the CFSSP algorithm and PEP2D algorithm. (**D**) Investigation of the secondary structure for Buc-VBM (amino acid 363–400) using CD spectroscopy. The Y-axis represents molar ellipticity ((θ)), which is corrected for the concentration. The X-axis represents the corresponding wavelengths. In the native aqueous conditions and neutral pH, the CD spectra calculated for Buc-VBM were typical for a disordered protein (very low CD spectra above 210 nm and negative spectra near 195 nm: black line). After the addition of different concentrations of the crowding agent TFE (35% red line and 50% blue line), Buc-VBM displays CD spectra characteristic for α-helices (negative CD spectra at 222 nm and 208 nm and positive spectra at 193 nm). (**E**) The C-score values predicted for the homology models of zfVasa by the I-Tasser program. (**F**) The model with the highest C-score (−0.87) was selected as the homology model for zfVasa. The RecA-NTD of zfVasa is colored in marine blue, while RecA-CTD is colored in salmon. (**G**) The structure solved for *Drosophila* Vasa (DmVasa). The structure is composed of the amino acids 200–623 (PDB ID 2DB3 [44]). RecA-NTD of DmVasa is colored in magenta, while RecA-CTD is colored in blue. (**H**) Superposition of the DmVasa and zfVasa structures using the PyMol tool shows that two structures have nearly identical secondary structures (root mean square deviation (RMSD) = 0.333).

Recent structural studies on *Drosophila* Oskar demonstrated that the C-terminal extension of the LOTUS domain (eLOTUS) adopts an α-helical structure during interaction with DmVasa [24]. Interestingly, we noticed that Buc-VBM (amino acids 363–400) also encodes a structurally disordered region based on computational predictions (Supplementary Figure S1). Thus, we hypothesized that Buc and Osk are likely to undergo the same structural transition during interactions with zfVasa, explaining their equivalent functions during germ cell specification [23]. As there is no structure for Buc known, we conducted in silico secondary structure predictions for Buc-VBM (amino acids 363–400) using three different algorithms: CFSSP, PEP2D, and Jnet, which are widely used in structural biology to predict protein secondary structures [40,41,42,43]. Three algorithms independently predicted two α-helices for Buc-VBM, which share similar amino acids (Figure 3A–C). The first α-helix (α1) consisted of glutamic acid (E), arginine (R), glutamine (Q), and serine (S), while the second α-helix (α2) consisted of arginine (R), aspartic acid (D), glutamic acid (E), and methionine (M) (Figure 3A-C). These results make the Buc-VBM an interesting object for further experiments to investigate its structure.

We applied far-UV circular dichroism (CD) spectroscopy to confirm the in silico predicted secondary structures of Buc-VBM. CD spectroscopy uses light absorption to measure the difference in absorbance of right- and left-handed circular polarized light when it passes through optically active molecules [45]. Predominantly, amide groups of the polypeptide backbone are optically active, and their CD spectrum changes with the local conformation of a secondary structure in the protein. Therefore, we calculated the molar ellipticity at native, aqueous conditions and a neutral pH ((θ)deg × cm^2^/dmol) for Buc-VBM. It showed a strong negative molar ellipticity at 200 nm and a low negative molar ellipticity at 210–230 nm and 190 nm (Figure 3D: black line). Thus, the CD spectrum of Buc-VBM is typical for a disordered protein that lacks any defined secondary structure and experimentally confirms the prediction of an intrinsically disordered region (IDR; Supplementary Figure S1).

Organic solvent trifluoroethanol (TFE) with its structure-inducing properties has been used to stabilize secondary structures in short peptides [46]. It is suggested that TFE disrupts hydrogen bond formation between water molecules in the solvent shell and the amide proton of the peptide backbone. Consequently, intramolecular hydrogen bonds are strengthened, and the stability of the secondary structure elements increases [47,48]. Using TFE, we tested the propensity of Buc-VBM to form stable secondary structures. Fascinatingly, we detected that Buc-VBM displays the typical CD spectra for α-helices in the presence of 35% (Figure 3D: red line), as well as 50%, TFE (Figure 3D: blue line). These results suggest that Buc-VBM is likely to adopt an ordered conformation from its disordered nature when it (partially) loses its hydration shell as occurs during protein–protein interactions. Taken together, our in silico secondary structure predictions and CD spectroscopy experiments provide strong support for the hypothesis that the Buc-zfVasa interaction shows structural similarities to the well-characterized eLOTUS-DmVasa complex in *Drosophila*.

### 3.4. Homology Modeling for zfVasa

Similar to the secondary structure prediction for Buc-VBM, we did homology modeling for zfVasa to gain in-depth information of the Buc-zfVasa interaction. Initially, we aligned DmVasa encoding the amino acids 200–623, whose crystal structure was already solved, with zfVasa encoding the amino acids 227–670 that we used in the GST pull-down assay [44] (Supplementary Figure S3). The alignment revealed that the secondary structures of zfVasa and DmVasa are highly conserved.

After that, we calculated the homology model for zfVasa using the I-Tasser program [49]. The I-Tasser program predicted four homology models. From there, we selected the model with the highest C-score (−0.87), as the highest C-score predicts the best model [49] (Figure 3E). The superimposition of the selected zfVasa structure with the published structure for DmVasa using PyMol software (PyMol; Version 2.2.3, Schrödinger, LLC. [44]) illustrated that the predicted secondary structures of zfVasa are nearly identical to the DmVasa structure (Figure 3F–H). Thus, we utilized the predicted zfVasa model as a base for the subsequent experiments.

### 3.5. Identification of Amino Acids in the Buc-VBM, Which Are Required for zfVasa Interaction

**Figure 4:**
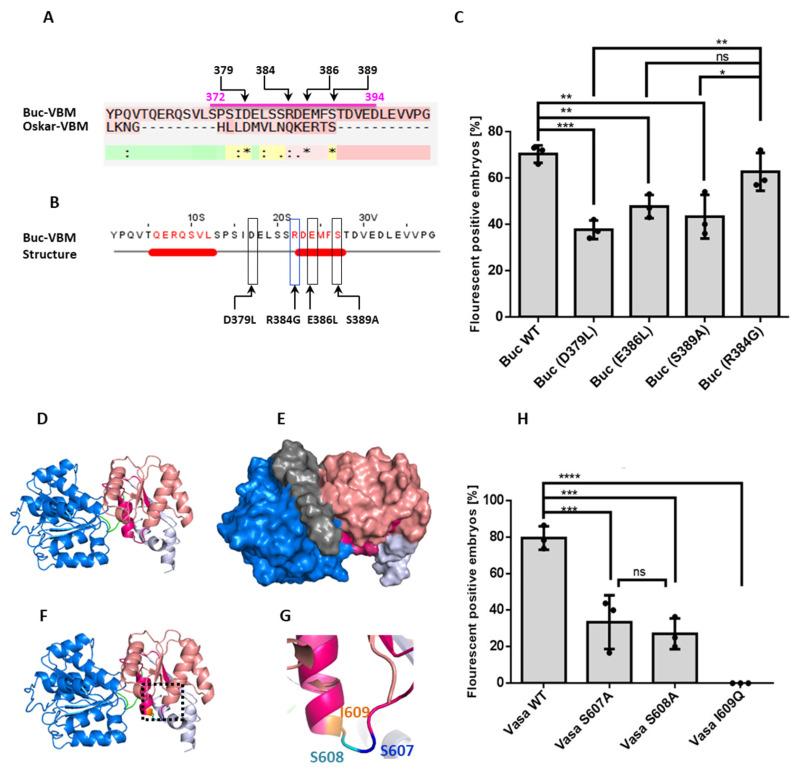
Isolation of the amino acid residues responsible for Buc and zfVasa interactions. (**A**) Alignment of the Buc-VBM with the Oskar eLOTUS domain. An asterisk (*) denotes the positions with a fully conserved residue. A colon (:) denotes conservation based on strongly similar properties—scoring > 0.5 in the PAM 250 matrix. A dot (.) denotes conservation based on weakly similar properties—scoring ≤ 0.5 in the PAM 250 matrix. An alignment of Buc-VBM and the eLOTUS domain revealed that D379, E384, and S386 are conserved between two sequences (black stars and positions of the amino acids are labeled on top of the sequence). (**B**) Representation of the three amino acids D379, E386, and S389 in JPred secondary structure prediction (black boxes) and amino acid R384 (blue box) used as a non-conserved control. (**C**) Quantification of mutant Buc construct interactions with zfVasa. The Y-axis represents the average fluorescent positive embryos. The X-axis represents the injected mutant Buc constructs. All the mutants, Buc D379L (36 ± 5.1%, *n* = 86), E386L (48 ± 9.4%, *n* = 77), and S389A (43 ± 3.4%, *n* = 50), showed a reduction of fluorescent embryos compared to wild-type Buc (70 ± 4.6%, *n*= 69) or in the control R384G (63 ± 8.2%, n = 63). In particular, the D379L mutant variant showed the weakest activity when compared to the Buc wild-type and control R384G. (**D**) Homology model predicted for zfVasa with the RecA-like N-terminal domain (blue) and RecA-like C-terminal domain (salmon). zfVasa-BBM is colored in magenta. (**E**) Surface representation of the predicted model showing the exposed region of zfVasa-BBM (magenta). (**F**) Model highlighting the exposed three amino acids (inset in the black dashed line) in the rainbow colors described in (**D**). (**G**) Magnification of the exposed amino acids S607 (blue), S608 (green), and I609 (orange). (**H**) Quantification of the mutant zfVasa construct interactions with Buc. The Y-axis represents fluorescent embryos from three independent experiments. The X-axis represents the injected mutant zfVasa constructs. zfVasa S607A (38 ± 14.0, *n* =61) and zfVasa S608A (27 ± 8.0, *n* = 33) showed a reduced number of fluorescence-positive embryos, whereas zfVasa I609Q completely lost the interaction (0 ± 0, *n* = 61) compared to the wild-type zfVasa (79 ± 6.4, *n* = 64). Moreover, there was no significant statistical difference observed between zfVasa S607A and S608A. Test statistics: Student’s *t*-test, * = 0.05, ** = 0.01, *** = 0.001, and **** = 0.0001. ns = not significant.

After the deletion of Buc-VBM (amino acids 372–394) from Buc and zfVasa-BBM (amino acids 600–625) from zfVasa, no interaction could be detected in vivo (Figure 1M,K). However, these deletion constructs might not fold into their native functional structure in the absence of these interaction motifs. Thus, finding point mutations in the Buc and zfVasa-binding interfaces that impair the interactions of both proteins would allow us to more precisely determine their interaction interface. It has been shown that the Osk-eLOTUS domain is necessary for DmVasa interactions and also forms an α-helix during the interaction with DmVasa [24]. To identify candidate amino acids, we aligned the peptide sequence of the Buc-VBM and the Osk-eLOTUS domains to analyze whether there are any conserved residues between these two peptides (Figure 4A). The alignment highlighted three amino acids of Buc-VBM: namely, aspartate (D379), glutamate (E386), and serine (S389), which are conserved between the two sequences (Figure 4A). When we mapped the position of these three residues onto the predicted secondary structures for Buc-VBM (Figure 3C), we found that D379 is part of a loop region connecting the two predicted α-helices. At the same time, E386 and S389 are part of the second α-helix (Figure 4B).

To study a putative functional role of these three amino acids during the interaction of Buc with zfVasa, we attempted to create a point in the mutations incl. D379L (aspartate to leucine), E386L (glutamate to leucine), and S389A (serine to alanine) in full-length Buc. However, the site-directed mutagenesis in full-length Buc failed. Thus, we generated these mutations in Buc-VBM (amino acids 363–400). As a control, we mutated a non-conserved amino acid R384G (arginine to glycine) (Figure 4B). After a co-injection of D379L, E386L, and S389A, together with zfVasa, we detected reduced fluorescence signals in the BiFC assay for all the mutants compared to wild-type Buc or the control Buc R384G mutant (Figure 4C).

Furthermore, among the three mutants tested, D379L exhibited the weakest activity (38%) compared to E386L (48.0%) and S389A (43.0%) (Figure 4C). Nevertheless, we did not observe activity differences among the three mutant variants (Supplementary Figure S5). Comparison of the three mutant variants against the control (R384G) also revealed that the D379L has the weakest activity (Figure 4C). Thus, these results suggest that D379 in Buc-VBM plays a major role for the interaction with zfVasa. Moreover, the predicted loop region of Buc-VBM contributes to forming the Buc-zfVasa-binding interface and the predicted second α-helix. Taken together, these data identify important amino acids in Buc that are critical for its interaction with zfVasa.

### 3.6. Amino Acids in zfVasa-BBM Required for Buc Interaction

We used the predicted zfVasa model as a template to investigate the key amino acid residues essential to interact with Buc. The predicted zfVasa model showed that the zfVasa-BBM consists of one α-helix and a β-sheet connected by a flexible loop (Figure 4D). Further, an analysis of surface accessibility of the predicted structure revealed that three amino acids, S607 (serine 607), S608 (Serine 608), and I609 (isoleucine 609), on the flexible loop are solvent exposed on the protein surface (Figure 4E–G). This supports a role for interacting with Buc. Therefore, we mutated these residues as S607A (serine to alanine), S608A (serine to alanine), and I609Q (isoleucine to glutamine) in full-length zfVasa. The S607A (38%) and S608A (27%) zfVasa mutants showed a markedly reduced interaction compared to the full-length Buc (Figure 4H). More importantly, the zfVasa I609Q mutant completely lost the ability to interact with Buc (Figure 4H). To test whether zfVasa I609Q decreases the thermodynamic stability of the zfVasa protein, we generated zfVasa variants fused with GFP (zfVasa-GFP). There, we overexpressed the same amount of RNA (200 ng/µL) of these zfVasa variants and quantified the fluorescence intensity after 3 hpf. In the results, all the zfVasa variants displayed similar expression levels in vivo as wild-type zfVasa-GFP (Supplementary Figure S4). These data are consistent with the conclusion that I609 is not required for the stability of zfVasa but is a critical residue for binding to Buc.

### 3.7. The Buc-VBM Is a Novel Activator of zfVasa ATPase Activity

Our data show that Buc directly binds to zfVasa. This raises the question of what the functional consequences of this interaction are. It has been previously demonstrated that protein cofactors modulate the enzymatic ATPase activity of DEAD box proteins. A recent study discovered that the Osk LOTUS domain stimulates the ATPase activity of DmVasa upon their interaction, albeit Osk does not belong to a canonical protein family of the ATPase regulating cofactors [24]. We next aimed to check whether Buc-VBM could modulate the ATPase activity of zfVasa upon binding. To quantify the zfVasa activity in vitro, we employed a spectrophotometric enzymatic assay monitoring nicotinamide adenine dinucleotide (NADH) conversion [33].

**Figure 5:**
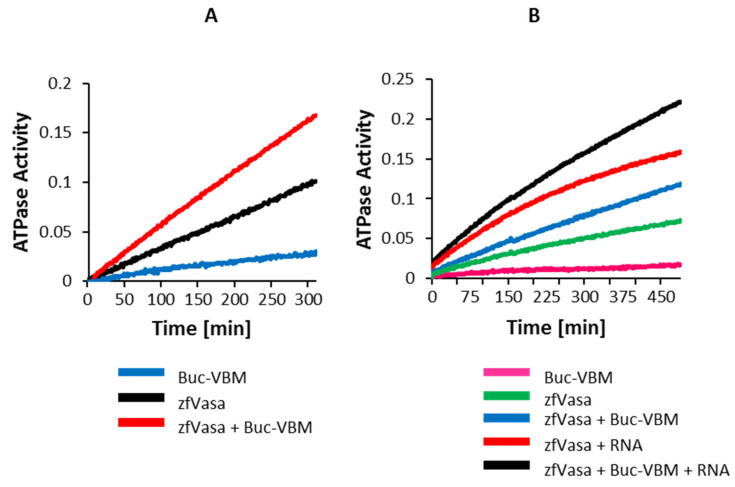
Buc-VBM activates Vasa helicase activity. (**A,B**) The Y-axis denotes the ATPase activity as a function of the reduction of NADH absorption at 340 nm. The X-axis denotes the incubation time in minutes. The data presented are averaged from three independent experiments. (**A**) The incubation of 100-µM Buc-VBM with 2.5-mM ATP showed very mild ATPase activity (negative control, blue line). A higher background ATPase activity was observed after the incubation of 5-µM zfVasa with 2.5-mM ATP (black line). Fascinatingly, the ATPase activity was approximately doubled when 100-µM Buc-VBM was added to 5-µM zfVasa and 2.5-mM ATP. (**B**) The incubation of 100-µM Buc-VBM with 2.5-mM ATP showed mild ATPase activity (pink line). The incubation of 5-µM zfVasa with 2.5-mM ATP displayed relatively higher ATPase activity (green line). The incubation of 5-µM zfVasa with 50-µM ssRNA showed enhanced ATPase activity (red line) compared to 5-µM zfVasa with 100-µM Buc-VBM (blue line). zfVasa ATPase activity was further enhanced in the presence of ssRNA and Buc-VBM (black line).

In principle, this assay monitors the reduction of NADH absorbance at 340 nm, which is directly proportional to the rate of ATP hydrolysis. In the control experiments, we observed a mild basal ATPase activity after incubating 5-µM zfVasa together with 2.5-mM ATP (Figure 5A: black line) compared to the samples incubated with only 100-µM Buc (Figure 5A: blue line). However, the ATPase activity was strongly induced upon the addition of 100-µM Buc-VBM to zfVasa (Figure 5A: red line). As the RNA-bound state of the DEAD box helicase exhibits increased activity, we next analyzed the ATPase activity of zfVasa in the presence of RNA. After the incubation of 50-µM ssRNA (polyA_(8)_) with zfVasa, we indeed observed an elevated ATPase activity compared to the sample without RNA (Figure 5B: red vs. green line). Fascinatingly, the ATPase activity was more potent in the presence of Buc-VBM and ssRNA compared to the zfVasa helicase core with ssRNA (Figure 5B: black vs. the red line). These results suggest that Buc-VBM functions as a novel cofactor that stimulates the ATPase activity of zfVasa. These results would therefore annotate the first biochemical function for the Buc protein as an ATPase activator.

### 3.8. The Buc-VBM and the zfVasa-BBM Inhibit PGC Formation in Zebrafish

**Figure 6:**
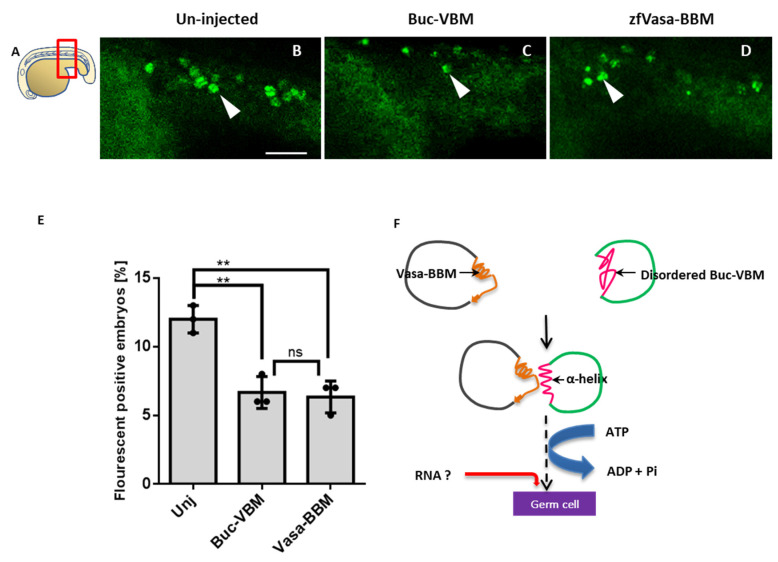
Buc-VBM and zfVasa-BBM inhibit germ cell specification in vivo. (**A**) Cartoon representation of the lateral view shown in panels (**B**–**D**) of 15–18-somite stage zebrafish larvae, with the animal to the left. Imaging area boxed in red. (**B**) Live un-injected embryos with fluorescent germ cells (green cells (white arrow); transgenic for Buc-GFP). (**C**) Embryos injected with Buc-VBM and (**D**) zfVasa-BBM. (**E**) Quantification of the germ cell number. Embryos injected with either Buc-VBM (6 ± 1.0, *n* =18) or zfVasa-BBM (6.3 ± 1.1, *n* = 16) showed a reduction of germ cells compared to the un-injected embryos (12 ± 1.6, *n* = 15). Additionally, we did not observe a significant statistical difference between Buc-VBM and zfVasa-BBM. The data presented are averaged from three independent experiments. The Y-axis represents the average germ cell count, and the X-axis represents the injected constructs Buc-VBM and zfVasa-BBM. Error bars represent the standard deviation of the mean. (**F**) Hypothetical model of the Buc-zfVasa interaction. Buc (green line) binds directly to zfVasa (black line) through zfVasa-BBM (orange) and Buc-VBM (pink). During the interaction, disordered Buc-VBM adopts an α-helical structure. Conversely, Buc-VBM stimulates the ATPase activity of zfVasa (blue arrow), which is further enhanced when RNA (red line) binds to zfVasa. The helicase activity probably triggers an unknown downstream RNA processing specifying primordial germ cell development. Scale bar 50 µm. Test statistics: Student’s *t*-test, ** = 0.01. ns = nonsignificant.

As Buc-VBM stimulates the ATPase activity of zfVasa, we hypothesized that the ATPase activity probably plays an essential role during germ cell specification. In this scenario, the overexpression of Buc-VBM in one-cell stage embryos would possibly induce more germ cells. To test this hypothesis, we injected the mRNA of Buc-VBM into one-cell embryos of Buc-eGFP transgenic fish. Somewhat unexpectedly, we observed a reduction in the number of germ cells in embryos injected with Buc-VBM compared to the controls at the 15–18-somite stage (Figure 6C,E). The reduced number of germ cells suggests a dominant-negative effect of Buc-VBM. This result rather supports a model in which Buc-VBM interacts with endogenous zfVasa, but this interaction is unable to recruit additional critical molecule/s and, thus, renders the complex inactive.

This model predicts that zfVasa-BBM would also generate the same inactive complex with endogenous Buc and eventually lead to a reduced number of germ cells. To test this hypothesis, we repeated the same overexpression experiments with zfVasa-BBM. Indeed, we observed a reduction in the germ cell numbers in embryos after injection with zfVasa-BBM, confirming that zfVasa-BBM also acts as a dominant-negative peptide (Figure 6D,E). Taken together, these data show that both binding motives alone are insufficient to form a fully functional germ cell specification complex. This is most likely due to the fact that other parts of the protein sequences could be necessary for the recruitment of additional cofactors required for germ cell development.

## 4. Discussion

Here, we characterized the interaction of the Buc-zfVasa core complex, which is required for germ cell specification in zebrafish. Our results provide evidence that the Buc-zfVasa interaction is not sufficient for germ cell specification. Thus, the Buc-zfVasa complex probably needs to recruit additional germplasm factors, such as, e.g., RNA molecules, to form a functional complex during germ cell specification. The recruitment of RNA to the complex triggered the ATPase activity of zfVasa, which, in turn, regulates the downstream functions in the germ cell specification pathway.

In this study, we isolated two novel interaction motifs, Buc-VBM (amino acids 372–394) and zfVasa-BBM (amino acids 600–625). Previously, we recognized that the sequence encoding amino acids 372–394 located within Buc-VBM (amino acid 363–400) is highly conserved among the Buc orthologs [23]. We have now discovered that this region is indeed responsible for the interaction with zfVasa. Interestingly, this sequence is also conserved in the chicken (*Gallus gallus)* homolog of Buc and in the *Xenopus* homolog of Buc, Xvelo. Further, the chicken Vasa homolog (Cvh) and the *Xenopus* Vasa-like gene 1 (XVLG1) are essential for the formation of PGCs [50,51]. Therefore, this conservation supports the hypothesis that other vertebrates with conserved Buc orthologs likely use a similar interaction with Vasa to specify germ cells.

The Osk-LOTUS domain physically binds to the RecA-CTD of DmVasa and stimulates the ATPase activity of DmVasa [24,25]. Notably, the C-terminal extension of the LOTUS domain (eLOTUS) adopts an α-helix (α5) from a structurally disordered state upon binding to DmVasa and is essential for DmVasa interactions [24]. A recent study has also discovered two novel LOTUS domain containing proteins—namely, the MEG-3 interacting protein (MIP)-1 and -2 in *C. elegans*—which are required for P-granule assembly and germ line maintenance. Remarkably, the predicted structure for the LOTUS domains of MIP-1 and MIP-2 also possess the α5-helix and physically bind to the Vasa homolog of *C. elegans*, GLH-1 [52]. Our in silico secondary structure prediction revealed that Buc-VBM has a propensity to form two α-helices. The second α-helix is completely embedded within the highly conserved sequence (amino acids 372–394) (Figure 3A–C). On the other hand, the CD spectroscopy data demonstrated that the Buc-VBM also tends to form α-helices from its disordered state in the presence of TFE (Figure 3D). Collectively, these data suggest that the Buc, MIPs, and Osk proteins share a similar three-dimensional structure during interaction with zfVasa, GLH-1, and DmVasa, which, in turn, explains the functional similarity between Buc and Osk.

To the contrary, the predicted homology model for zfVasa illustrates that zfVasa-BBM comprises one α-helix, one β-sheet, and a flexible loop, which connects these two elements. Mainly, we show that residue I609 of zfVasa located within the flexible loop is a key residue in zfVasa-BBM (amino acids 600–625) that controls the Buc-zfVasa interaction. Therefore, the homology model for zfVasa shows that the zfVasa-BBM motif is structurally different from the Osk-binding motif in DmVasa, which is an α-helix. Strikingly, the study on MIPs–GLH-1 interactions revealed that their interaction interface is markedly different from the LOTUS–DmVasa interaction interface [52]. It seems that zfVasa, GLH-1, and DmVasa occupy a pliable species-specific interaction interface for Buc, MIPs, and Osk to perform their unique biochemical activities in the PGC specification pathway. However, an in-depth understanding of the Buc and zfVasa interaction is difficult due to the lack of structural data. Therefore, the co-crystallization of recombinant Buc-VBM together with zfVasa would provide better insight to understand the Buc and zfVasa interaction. We started the co-crystallization of Buc-VBM (amino acids 363–400) and zfVasa (amino acids 227–670) but, to this end, could not get crystal growth despite many trials.

Furthermore, we identified three functionally important residues: D379, E386, and D389 that are conserved in Osk-eLOTUS and Buc-VBM. Of these amino acids, E386 and E389 are located in the second α-helix (Figure 3A–C), while D379 is in the predicted flexible loop region. A mutation of these amino acids reduced the strength of the interaction with zfVasa. Although R384 is located in the second α-helix, an R384G mutation showed a similar interaction pattern as wild-type Buc-VBM. Therefore, we hypothesized that D379, E386, and E389 collectively create the zfVasa interaction interface, while R384 does not contribute to the zfVasa interaction positioning, its side chain opposite to the interaction interface.

Like shown for the Osk-LOTUS domain, our current results indicate that the Buc-VBM acts as a novel cofactor that stimulates the ATPase activity of zfVasa. This discovery annotates the first biochemical function for Buc as a zfVasa ATPase activator. Thus, our results also clarify a hitherto open question regarding the role of Vasa in germline development. In some organisms, such as *Drosophila*, DmVasa expression is restricted to the germline. By contrast, zfVasa is ubiquitously expressed in early embryogenesis and only restricted to the germline during later embryogenesis [23]. According to our data, not the expression level but, rather, the enzymatic activity of zfVasa is a molecular marker for the prospective germline. Unfortunately, the product of “active” Vasa so far remains unknown. We therefore suggest that the expression or localization of zfVasa activators like Buc or Oskar are currently the best molecular markers to predict the future germ cells of an embryo. Unfortunately, Buc and Oskar seem to be restricted to a few species. Additionally, both Buc and Oskar are predicted to be IDPs [23]. Therefore, the identification of homologous proteins in other species is difficult, as these IDPs evolve very fast, masking conserved sequence motifs [4].

So far, the implications of the zfVasa ATPase activity in the PGC specification pathway are unknown. Identifying a point mutation that impairs the zfVasa ATPase activity would provide a possible approach for addressing this question. It has been shown that the T546A mutation abolishes the ATPase activity of DmVasa [44]. Interestingly, we detected that the same amino acid is conserved (T585) in zfVasa. The overexpression of zfVasa T585A mutant in the germ cell induction assay revealed that the ectopic germ cell formation is similar to wild-type zfVasa [53]. Nevertheless, the same mutation analysis performed in GLH-1 detected fertility defects in *C. elegans* [54]. Therefore, the ATPase activity of zfVasa might not directly regulate germ cell specification, or the mutated residues probably have species-specific functions. Alternatively, this mutation might create a hypomorph that has sufficient activity in zebrafish overexpression experiments to specify germ cells. However, the impact of these mutants has not been studied in vivo [44]. Thus, additional experiments are necessary to confirm that the T585A mutation retains no ATPase activity in vivo.

We show that overexpressed Buc-VBM and zfVasa-BBM in one-cell stage embryos act as dominant-negative peptides during germ cell specification, suggesting that another critical molecule/s is missing in the germ cell specification pathway. Previous studies have shown that Oskar binds to nanos RNA in vivo [25]. The *Xenopus* homolog of Buc, Xvelo, showed that recombinant protein recruits RNA when it forms a Xvelo network in vitro [55]. We also showed that Buc co-localizes with nanos RNA during oogenesis and embryogenesis [22]. Recently, we demonstrated that full-length Buc (amino acids 1–639) interacts with nanos 3–3′ UTR in vitro [23]. Interestingly, a Buc mutant, BucP^106^ (amino acids 1–601), did not induce PGCs in the germ cell induction assay, even though it contained the Buc-VBM [23]. Hence, this suggests that the 38 C-terminal amino acids of Buc are essential for PGC specification. Analyses of the physicochemical properties revealed that the terminal region is positively charged, as this region is rich in arginine and lysine residues. Therefore, we speculate that this region possesses a potential RNA-binding motif. Alternatively, it has been shown that the C-terminus of Buc recruits Tdrd6a [27]. Additionally, in our results, we show that the ATPase activity of zfVasa is increased in the presence of RNA, providing anther argument supporting that RNA is the critical third component.

Collectively, we hypothesize that RNA and/or an unknown interaction partner of Buc creates a core protein complex, including Buc and zfVasa, necessary for germ cell specification. Therefore, the lack of specificity would probably explain the reduction of the germ cells upon the overexpression of Buc-VBM and Vasa-BBM. Thus, these peptides might not interact with RNA in vivo; instead, these peptides might lock the Buc-zfVasa complex into an inactive state. Therefore, mapping the RNA-binding motif in Buc and an investigation of the role of other Buc-binding proteins such as Tdrd6 and Igf2bp3 in the Buc-zfVasa complex will provide more detail to understand the role of Buc in germ cell specification.

## Data Availability

The data supporting the findings of this study are available from the first author upon request.

## Supplementary Materials

The following are available online at https://www.mdpi.com/article/10.3390/biom11101507/s1: Figure S1: Buc and zfVasa are intrinsically disordered proteins. Figure S2: Vasa-BBM is potentially located between amino acids 600–665. Figure S3: Amino acid sequence alignment of Drosophila and zebrafish Vasa. Figure S4: The I609Q mutation did not reduce Vasa stability. Figure S5: Comparison of the Buc mutant variants.
