# Supplementary material for: Bucky Ball Is a Novel Zebrafish Vasa ATPase Activator
Source: Biomolecules. 2021 Oct 13;11(10):1507. doi: 10.3390/biom11101507 (PMC8533965; doi:10.3390/biom11101507)
Supplement: Supplementary file 1 [file biomolecules-11-01507-s001.zip › biomolecules-1406750-supplementary.pdf]

Supplementary Figures

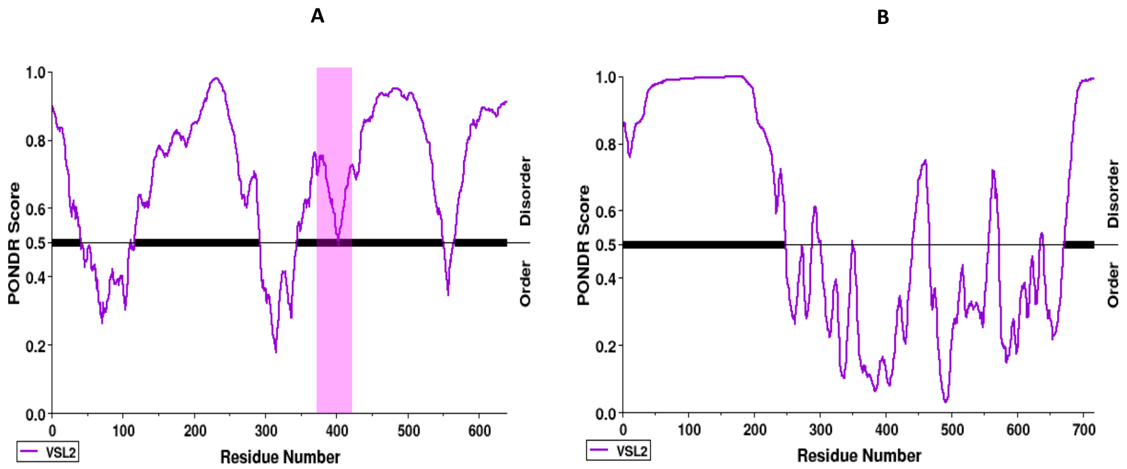

Supplementary Figure S1. Buc and zfVasa are intrinsically disordered proteins**.** Intrinsically disordered properties of Buc (A) and zfVasa (B) predicted using PONDR protein disorder predictor using VSL2 algorithm. The Y-axis represents the generated disordered PONDR score. The X-axis represents the number of amino acids for a given protein. Threshold value between ordered and disordered protein considered as 0.5 (thick horizontal black line). Distribution of the disordered and ordered properties are represented by the purple line. Protein regions above the threshold value are ordered while regions below the threshold value are disordered. The Buc-VBM (amino acid 363-400) is highlighted with a pink box.

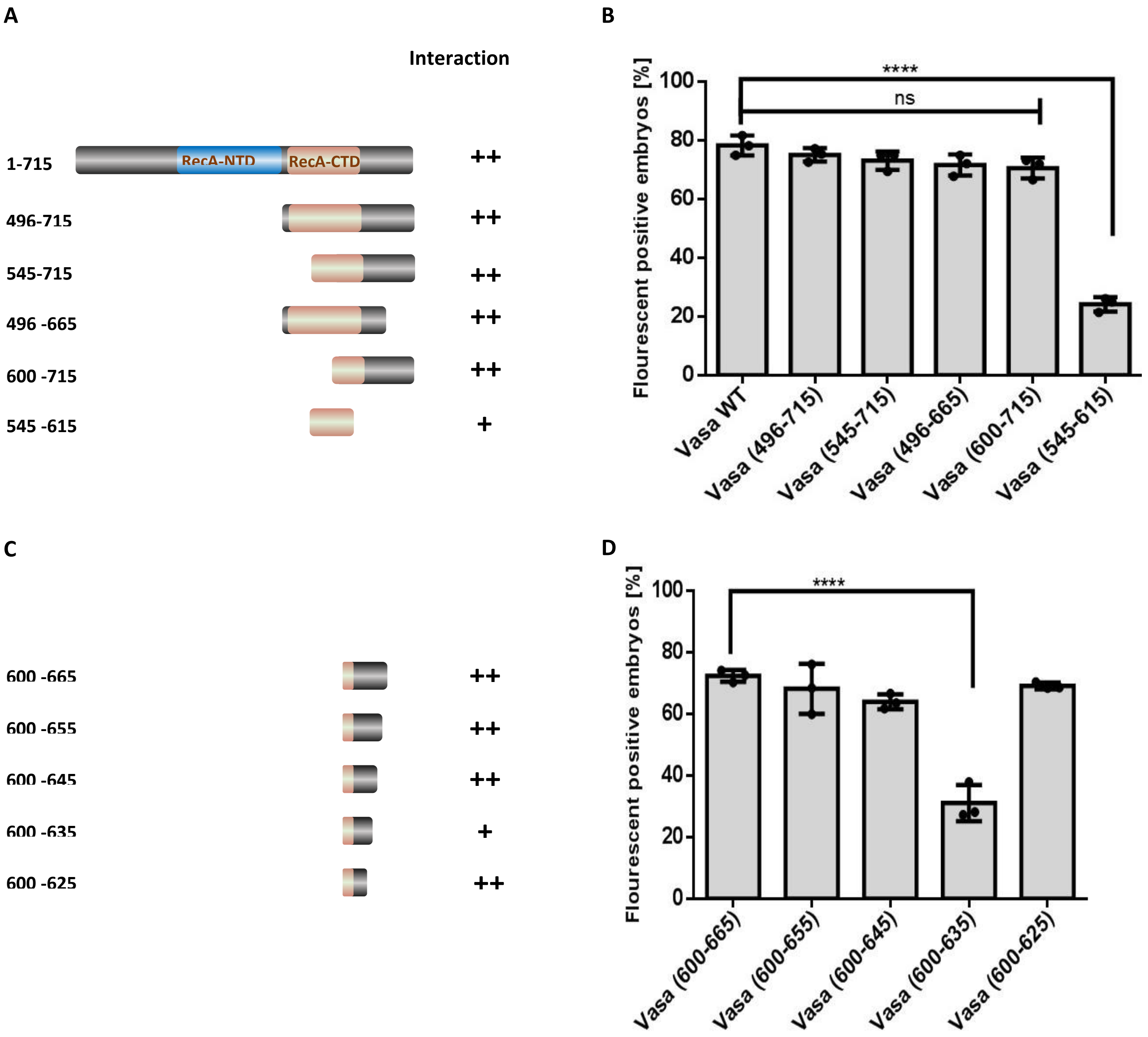
A
Interaction
1-715
RecA-NTD
RecA-CTD
++
496-715
++
545-715
++
496 -665
++
600 -715
++
545 -615
+
B
Flourescent positive embryos [%]
100
80
60
40
20
0
****
ns
Vasa WT
Vasa (496-715)
Vasa (545-715)
Vasa (496-665)
Vasa (600-715)
Vasa (545-615)
C
600 -665
++
600 -655
++
600 -645
++
600 -635
+
600 -625
++
D
Flourescent positive embryos [%]
100
80
60
40
20
0
****
Vasa (600-665)
Vasa (600-655)
Vasa (600-645)
Vasa (600-635)
Vasa (600-625)

Supplementary Figure S2. zfVasa-BBM is potentially located between amino acid 600-665. (A) Schematic illustration of systematic truncations of zfVasa (dark grey) with the helicase core containing N-terminal (blue) and C-terminal (light rose) RecA-like domains. Numbers left to the colored bars indicate the corresponding amino acids. (B) Quantification of fluorescent embryos after microinjection of mRNA with BiFC Buc and zfVasa constructs. The data presented are averaged from three independent experiments. The X-axis represents percentage of average fluorescent positive embryos and the Y-axis represents injected constructs. Error bars represent standard deviation of the mean. Fluorescent positive embryos ≥ 60% denoted as '++' and fluorescent positive embryos ≤ 60% shown as '+'. All zfVasa constructs were co-injected with wild-type Buc. Wild-type zfVasa (positive control: 78±3.4%, n= 61), zfVasa [496-715]; (75±3.4%, n= 85), zfVasa [545-715]; (73±3.1%, n= 63), zfVasa [496-665]; (71±3.6%, n= 58) and zfVasa [600-715]; (70±3.5%, n= 58) constructs show ≥ 60% fluorescent embryos, while zfVasa [545-615]; (24±2.4%, n= 53) shows ≤ 40% fluorescent embryos. (C) Schematic illustration of systematic truncation of zfVasa (dark grey) with the helicase core containing N-terminal (blue) and C-terminal (light rose) RecA-like domains. Numbers left to the colored bars indicate the corresponding amino acids. (D) Quantification of fluorescent positive embryos based on the different combination of BiFC Buc and zfVasa constructs. The data presented are averaged from three independent experiments. The X-axis represents the percentage of fluorescent embryos, and the Y-axis shows the injected constructs. Error bars represent standard deviation of the mean. Average fluorescent positive embryos ≥ 60% denoted as '++' and average fluorescent positive embryos ≤ 60% shown as '+'. All the zfVasa constructs were co-injected with wild-type Buc. There, zfVasa [600-665]; (72±1.9%, n= 80), zfVasa [600-655]; (68±8.0%, n= 81) and zfVasa [600-645]; (64±2.4%, n= 67) show ≥ 60% fluorescent embryos while zfVasa [600-635]; (31±5.9%, n= 90) shows ≤ 40% fluorescent embryos. Interestingly, zfVasa [600-625]; (69±8.2%, n= 64) shows ≥ 60% fluorescent embryos. Test statistics – Student's t-test. **** = 0.0001. ns = non-significant.

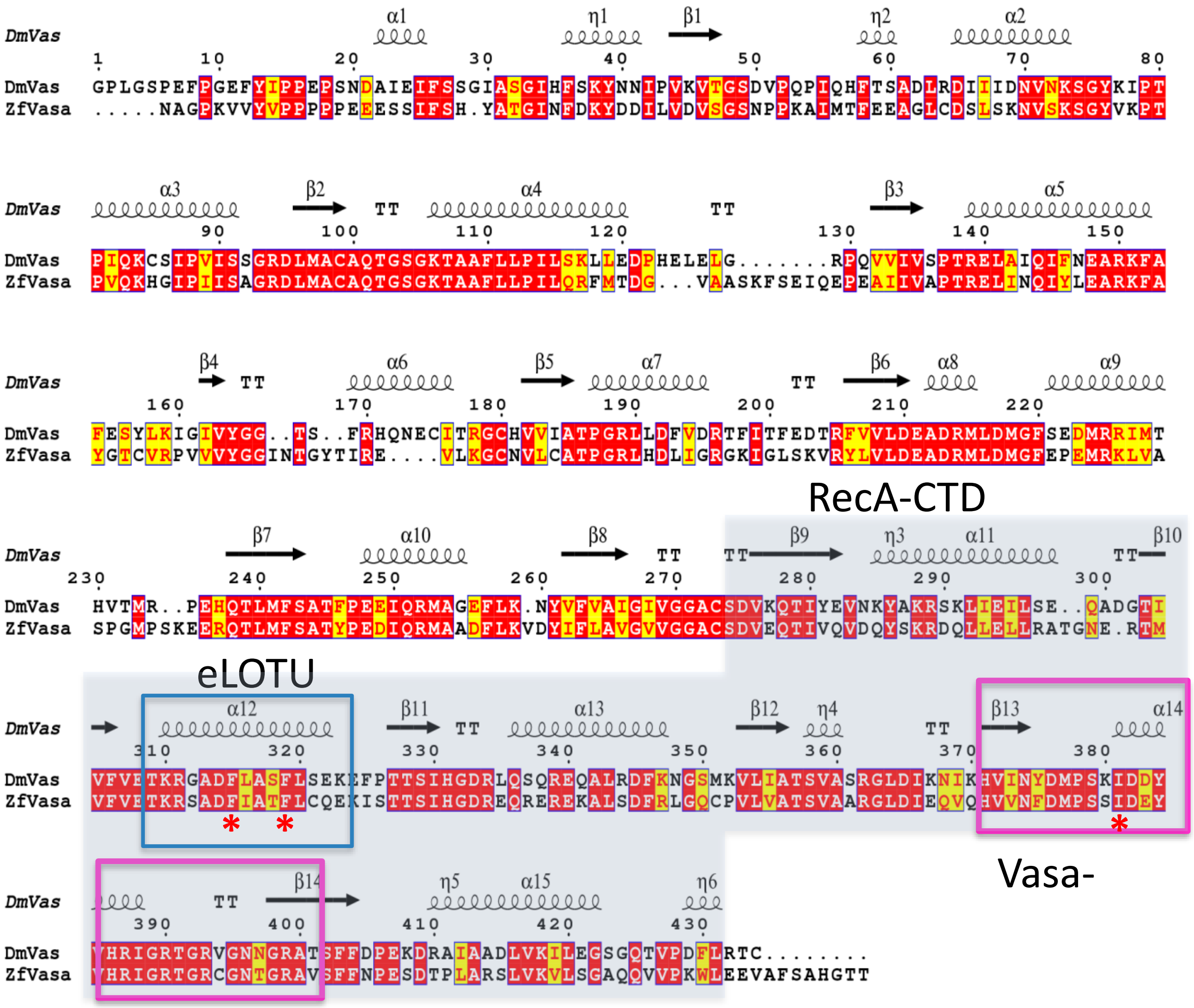

Supplementary Figure S3. Amino acid sequence alignment of *Drosophila* and zebrafish Vasa**.** Protein sequences were aligned using the T-Coffee multiple alignment server [33]. For the alignment, we used partial sequences of Drosophila Vasa (DmVas; amino acids 200-623), whose structure is already solved, and zebrafish Vasa (ZfVasa; amino acids 227-670), which we used for purification. Identities are boxed in red, similarities of physico-chemical properties are boxed in yellow. Secondary structures indicated above the alignment were predicted using Espript 3.0 based on the Drosophila crystal structure PDB ID: 2DB3 [44]Spirals indicate α-helices and arrows indicate β-sheets. Conserved RecA-CTD colored in grey. The α-helices, which binds to eLOTUS domain boxed in blue while the Buc binding motif is boxed in pink. The conserved amino acids between DmVasa and zfVasa: F504, F508 of DmVasa (F504: red star and F508: green star in the blue box) and I609 of zfVasa (blue star in the pink box).

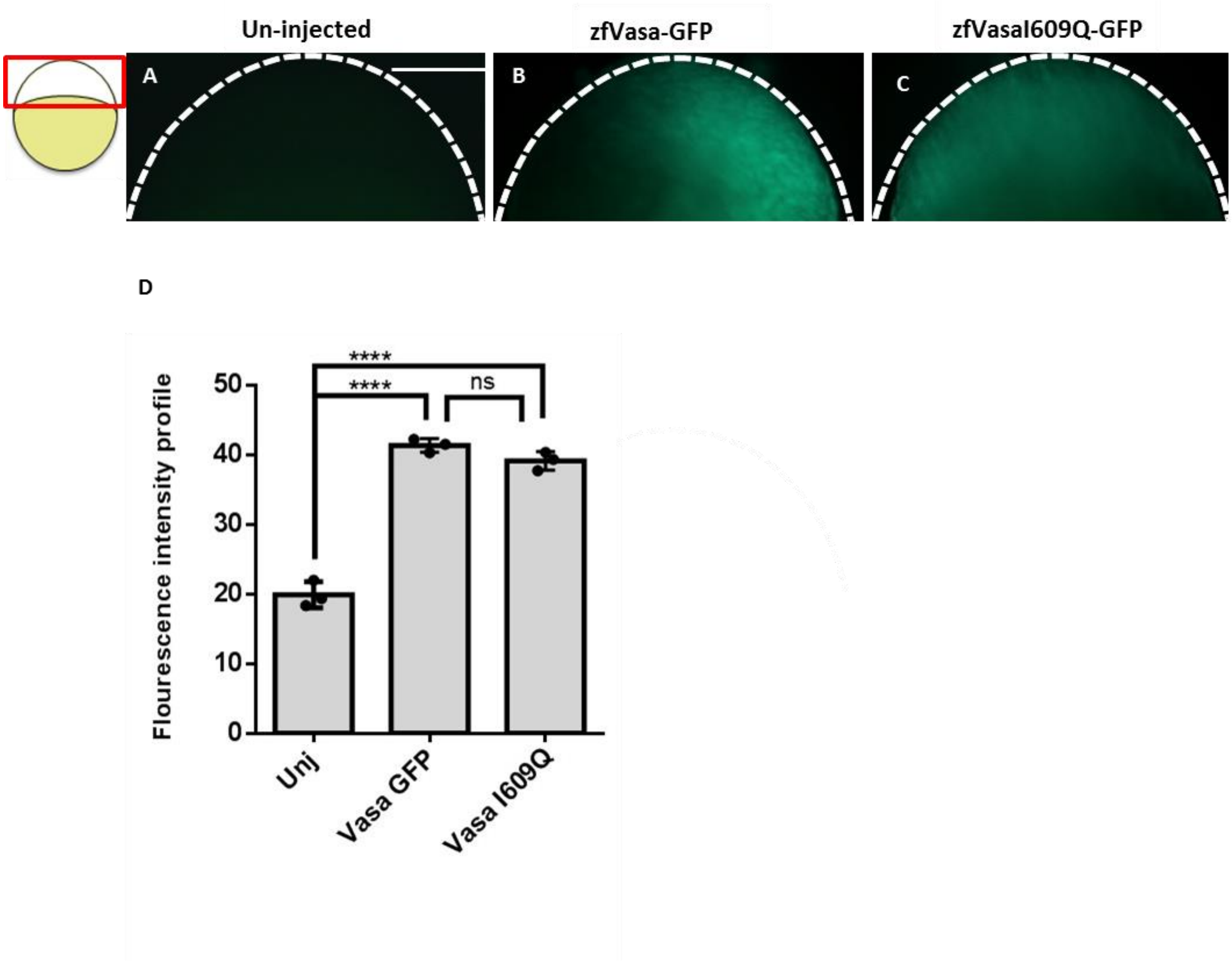

Supplementary Figure S4. The I609Q mutation did not reduce Vasa stability. (A-C) Confocal images of live embryos at 3 hpf after injection of zfVasa constructs. The imaging area is boxed in red as indicated in the cartoon on the left. This region is outlined with a white dashed line in the panel (A-C). (A) Un-injected embryos (B) Embryo injected with zfVasa-GFP and (C) Embryos injected with zfVasa I609-GFP (D) Quantification of fluorescent signal of injected embryos. The Y-axis represents the average fluorescence intensity while the X- axis represents the injected constructs. Embryos injected with zfVasa-I609Q-GFP showed a similar fluorescence intensity (39±0.86, n =3) in the blastodisc as wild-type zfVasa-GFP (41±0.69, n =3) when compared to an un-injected control (22±3.2, n =3) thus, suggesting that the zfVasa I609Q mutation did not disturb protein expression or stability. Similarly, we did not observe difference of fluorescence intensity between zfVasa-GFP and zfVasaI609Q-GFP. Test statistics – Student's t-test. **** = 0.0001. ns = non significance. Scale bar 200 μm.

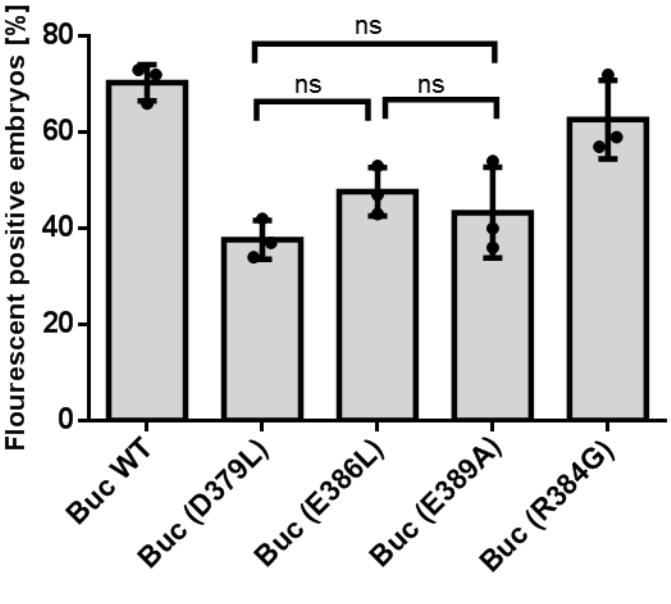

Supplementary Figure S5. Comparison Buc mutant variants. This graph is a continuation of graph included in Figure 4C. The Y-axis represents the average fluorescent positive embryos while the-axis represents the injected constructs. Analysis of variance (ANOVA) revealed that there is no statistical difference among the three Buc mutants: D379L, E386L and E389A. ns = non significant. Error bar represents standard deviation of the mean.
